# The Influence of Humidity on Assessing Irritation Threshold of Ammonia

**DOI:** 10.1155/2016/6015761

**Published:** 2016-06-09

**Authors:** Christian Monsé, Kirsten Sucker, Frank Hoffmeyer, Birger Jettkant, Hans Berresheim, Jürgen Bünger, Thomas Brüning

**Affiliations:** Institute for Prevention and Occupational Medicine of the German Social Accident Insurance, Institute of the Ruhr-University Bochum (IPA), Bürkle-de-la-Camp-Platz 1, 44789 Bochum, Germany

## Abstract

A large number of occupational exposure limit values (OELs) are based on avoiding of sensory irritation of the eyes and the upper respiratory tract. In order to investigate the chemosensory effect range of a chemical, odor and sensory irritation thresholds (lateralization thresholds, LTs) can be assessed. Humidity affects olfactory function and thus influences odor thresholds; however, a similar effect has not been shown for sensory irritation thresholds. The purpose of the present study was to explore whether LTs for ammonia vapor vary depending on the water vapor content of the inspired stimulus. Eight healthy nonsmoking volunteers were simultaneously exposed to ammonia vapor through one nostril and clean air through the other and were asked to determine which nostril received the chemical. Within experimental runs, ascending ammonia concentrations (60–350 ppm) that were either dry or humidified were administered at fixed time intervals. Geometric mean LTs obtained at wet (181 ppm) or dry (172 ppm) conditions did not differ significantly (*P* = 0.19) and were within the range of those reported by previous studies. These results suggest that humidity is not a critical factor in determining sensory irritation thresholds for ammonia, and future studies will examine if these findings are transferable to sensory irritation thresholds for other chemicals.

## 1. Introduction

A large portion of chemical substances, for which occupational exposure limits have been established, are based on avoiding of sensory irritation (Dick and Ahlers [1]; Edling and Lundberg [2]; Gaffney and Paustenbach [3]). The typical sensations of irritation—coolness, warmth, or sharpness—are mediated by the interaction of the chemical with receptors of the nervous system (e.g., free nerve endings of the trigeminal nerve) and can trigger defense mechanisms and reflexes (e.g., sneezing) (Doty et al. [4]; Morris and Shusterman [5]).

Psychophysical approaches that, for example, contribute to the determination of odor and sensory irritation thresholds may provide information on the irritant potency of a chemical. Importantly, odor and sensory irritation thresholds are usually measured for very short-term exposures and therefore cannot be used to extrapolate effects found in longer-term (i.e., occupational) exposures. Nevertheless, determining the chemosensory effect range reveals concentration-related transitions from olfactory to trigeminal stimulation and the particular ranges of concentrations where these changeovers take place, which is unknown for many chemicals (van Thriel et al. [6]). According to the model proposed by Shusterman [7], substances with sensory irritation thresholds lower than odor thresholds can be classified as potent irritants. Compounds with the odor thresholds slightly below the irritation thresholds have an intermediate irritant potency, and substances with a wide range between the odor and the sensory irritation thresholds are weak irritants but potent odorants. Data on the chemosensory effect range can further be used to design human whole-body exposure chamber studies or to evaluate existing studies reporting on sensory irritation [6]. By determining the chemosensory effect ranges for 15 sensory irritants and comparing the medians and the 5th and 95th percentile range with the German MAK values (“maximale Arbeitsplatz-Konzentration”: maximum workplace concentration), van Thriel et al. [6] demonstrated that the MAK values for dimethylamine, cyclohexylamine, and formic acid were below the median of the odor thresholds and thus slightly underestimated. Conversely, the MAK values for hydrochloric acid and ethyl acrylate were close to the median of the sensory irritation thresholds and as a result slightly overestimated.

Threshold testing is limited, because it is affected by the variability of human chemosensory sensation. Reasons for this variability arise from many factors, such as age, health, and experience, as well as differences in experimental methodologies. The field of odor threshold testing has evolved considerably in recent years, and improvements in methods and instrumentation as well as standardization efforts have significantly helped to overcome these drawbacks (CEN [8]; ASTM [9]). Hence, in olfactometric studies many of the factors that could affect odor threshold testing, for example, temperature, barometric pressure, or humidity, are carefully controlled. Using climate chambers, Kuehn et al. [10] were able to show that odor thresholds were lower at humid compared to dry conditions. Whether humidity also influences sensory irritation thresholds in humans has not yet been investigated. Only one previous study in rodents showed that the sensory irritation potency of ammonia is not influenced by inhaling wet vapor (with or without aerosol). In this study, Li and Pauluhn [11] exposed OF1 mice and Wistar rats to 131 to 1,776 ppm ammonia for 45 minutes via inhalation and examined their airway reflexes by the changes in respiratory patterns elicited by ammonia in either dry, steam-humidified, or aqueous aerosol containing vapor.

The aim of the present study was to assess the influence of humidity on sensory irritation thresholds in humans. As a result, lateralization thresholds of ammonia were measured using dynamic olfactometry to present different concentrations of ammonia in dry and wet air in a forced-choice procedure to one nostril and a blank stimulus simultaneously to the other nostril. This method is based on the ability to correctly localize trigeminal stimuli to the stimulated nostril, whereas olfactory stimuli cannot be lateralized (Kobal et al. [12]). This sensory irritation threshold is also referred to as the “lateralization threshold (LT).” Therefore, in order to compare the results of the present study with that of the above-mentioned animal study [11], we used the single chemical compound, ammonia (NH_3_).

Several recent publications have listed LTs for ammonia. For example, nasal LTs ranging from 37 to 67 ppm (Wise et al. [13]) and 167 to 179 ppm [14] have been reported, in addition to the study by van Thriel et al. [6] reporting the highest median LT for ammonia at 314 ppm. Different olfactometry methods resulted in mean LTs of 31.7 ppm for the static and 60.9 ppm for the dynamic method (Smeets et al. [15]). Furthermore, in a previous study using ammonia and a slightly different apparatus (Monsé et al. [16]), LTs were measured in the range of 109–208 ppm. Thus, in view of the variety of LT studies using ammonia, this compound seemed appropriate for the present purposes.

## 2. Material and Methods

### 2.1. Apparatus

A calibration gas generator (HovaCal 321/2-SP, IAS GmbH, Germany) was used to generate gaseous atmospheres. The device works with three different mass flow controllers (MFC 1, MFC 2, and MFC 3) which dose pure ammonia and compressed air (flow rates: MFC 1: 0.5–10 L/min, MFC 2: 2.5–50.0 L/min, and MFC 3: 1.0–10.0 mL/min). Water vapor was generated by evaporating water with a heating block operated at 130°C, which was subsequently pumped into the device with two computerized high precision syringes. The capacity of the 250 *μ*L syringes ranged from 8.0 *μ*L/min to 1.5 mL/min (for details, see [16]). All test sessions were conducted in an air-conditioned laboratory at IPA in Bochum, Germany (Monsé et al. [17]), with the mean temperature set to 21.0 ± 0.5°C.

### 2.2. Generation of Dry Ammonia Vapor

Pure ammonia from a compressed gas cylinder with a purity of 99.98% (Air Liquide Deutschland GmbH, Germany) was mixed with dry compressed air with a flow rate of 8 L/min to generate dry ammonia vapor (stimulus). Ammonia was handled with MFC 3. Compressed air was used as the carrier gas and generated from an oil-free air compressor (Medical Air Compressor MAC 200, Dräger Medical ANSI GmbH, Germany). Water vapor was eliminated by an integrated absorption dryer. Furthermore, activated coal and a fine (1 *μ*m of pore width) as well as a finest filter (0.01 *μ*m of pore width) were used to eliminate odorous contaminants and particles. Traces of humidity in the compressed air were analyzed by gas chromatography (Micro GC 3000, Agilent Technologies Inc., USA). A five-point calibration curve of different water vapor concentrations in the carrier gas (1,000, 2,500, 5,000, 7,500, and 10,000 ppm) was performed (data not shown). Each concentration was measured three times. The remaining water vapor concentration was 2,420 ppm (1.82 mg/m^3^ at 20°C, relative humidity (RH): 10.5%), calculated by performing a linear regression (*y* =* ax* +* b*;* a* = 114.99,* b* = 279,000; goodness of fit (*r* ^2^): 0.999) and raising the offset of the linear fit. The corresponding dew point was −12°C, calculated by using the software “Free Professional Humidity Calculator” (http://www.humidity-calculator.com).

### 2.3. Generation of Wet Ammonia Vapor

Pure ammonia was mixed with humidified air. The water vapor concentration was 22,420 ppm (16.8 mg/m^3^ at 20°C, RH: 97%) and the resulting dew point was 19.1°C which corresponds to almost 100% water saturation in air. Complete water saturation (RH: 100%) was given at 23,730 ppm at 20°C (17.8 mg/m^3^). Higher water vapor concentrations were avoided due to condensation effects in parts of the device. Ammonia concentrations were measured using a calibrated photo acoustic detector (Field Gas-Monitor 1412, Innova AirTech Instruments, Denmark). The detector was calibrated with a test gas, containing 500 ppm ammonia in nitrogen (Westfalen AG, Germany). Measurement accuracy was tested at 100 ppm and was in the range of ± 1.5% for wet and dry ammonia vapor.

### 2.4. Continuous Stimulus Generation

**Figure 1:**
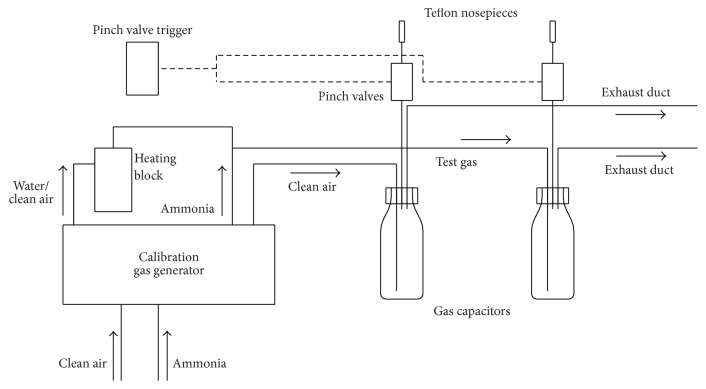
Schematic view of the lateralization threshold device.

The calibration gas generator described above was used to generate two parallel air flows (8 L/min), one with either dry or wet ammonia and one with clean air (Figure 1).

Two gas capacitors (250 mL glass bottles) were used to present sufficient amounts of ammonia and clean air to the nostrils. Each bottle was provided with a Teflon (4 mm outer and 2 mm inner diameter) tube ending into a closely fitting Teflon nosepiece (12 mm diameter). Two compressed air actuated pinch valves (connection size DN 6 with silicone tubes, KVT GmbH, Germany) between the gas capacitors and the Teflon nosepieces allowed for time controlled dosage of the stimuli by pulse-triggered opening and closing of the valves. Stimulus duration was set to two seconds. During the interstimulus interval (at least 30 s) when the valves were closed and the concentration step was prepared, gas excess was transported in an exhaust duct with a large tube (8 mm outer diameter).

### 2.5. Subjects

Eight healthy—five males and three females—nonasthmatic volunteers, aged between 35 and 50 years, were tested in order to assess the influence of humidity on the irritation threshold of ammonia. For females, pregnancy was excluded. In a screening period, volunteers completed a medical and psychological questionnaire, and a clinical examination was performed. Exclusion criteria were any tobacco smoking in the past year, and subjects with gastroesophageal reflux disease, history of asthma, allergic rhinitis, or nasal illness (e.g., nasal polyps or pronounced anatomical deviation) were not included. All participants had no history or reported symptoms of an upper respiratory infection six weeks prior to testing. Each subject had her or his rhinomanometry and pulmonary function measured before undergoing nasal ammonia exposures. The Medical Ethics Committee of the Ruhr-University Bochum approved the protocol for the study. Subjects gave written informed consent to participate. All experimental work was conducted in accordance with the Declaration of Helsinki.

### 2.6. Procedure

At the beginning of a trial, the test operator placed one nosepiece in the subject's right hand to be placed into the right nostril and the other nosepiece in the subject's left hand which had to be placed into the left nostril. Subjects were blindfolded using blackened eyeglasses, in order not to identify the nosepiece that offers the stimulus. The nostril that received ammonia varied randomly between the trials, and subjects were asked by the test operator to actively take a sniff after stimulus onset. After stimulus offset, the subjects were then asked to identify the nostril in which they received the ammonia, including their level of certainty. Three options were given: guess, doubt, and certain, and subjects did not receive any feedback as to the correct answer. Before the final data collection began, the subjects received an explanation of the lateralization task and had the opportunity to practice several times beforehand. Stimulus duration was fixed to 2 seconds and at least 30 seconds elapsed between successive trials.

According to the ascending method of limits described in [8], each run ended after two consecutive correct and certain responses, with most runs requiring about six trials. The individual threshold was calculated by averaging the first correctly detected and the last not correctly detected concentration (geometric mean).

Subjects were tested repeatedly on three nonconsecutive days. They completed four runs in a day, and at least 1 min separated successive runs. On each day, the first run started with 100 ppm ammonia, followed by 150 ppm. Consecutive concentrations were always increased by 50 ppm, whereas the start concentration of the next three runs varied (day #1: 70 ppm, 80 ppm, and 90 ppm; day #2: 60 ppm, 70 ppm, and 80 ppm; day #3: 70 ppm, 80 ppm, and 90 ppm). Additionally, the humidity of the ammonia airflow alternated between two runs (day #1: dry, wet, dry, wet; day #2: wet, dry, wet, dry; day #3: dry, wet, dry, wet). Hence, each individual threshold for wet and for dry ammonia was calculated based on six runs.

**Figure 2:**
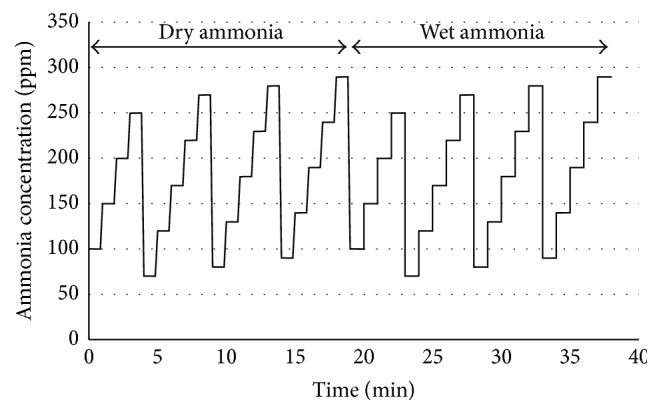
Presentation of four series of ascending dry ammonia concentrations and four series of ascending wet ammonia concentrations within two test sessions on test day #1.

Figure 2 shows an example of the presentation of ammonia atmospheres. Four series of ascending ammonia concentrations were performed, followed by four series of ascending ammonia concentrations while alternating changes in humidity after each series.

### 2.7. Statistic

The D'Agostino and Pearson omnibus normality test was used to assess value distribution. LTs were calculated as geometric mean (GM) with corresponding lower and upper 95% confidence interval (CI). Comparisons of paired data were performed with paired* t*-test. Correlations between thresholds for dry (LT_dry_) or wet (LT_wet_) ammonia were calculated with Pearson's test (*r*_P_). Differences between the two humidity conditions were illustrated in terms of Bland-Altman plots. The Bland-Altman method [18] calculates the mean difference between two methods of measurement (the “bias”) and 95% limits of agreement as the mean difference (2 SD). Data were analyzed using GraphPad Prism version 5.01 for Windows (GraphPad Software, San Diego, California, USA). A two-sided significance level of 0.05 was chosen for all tests.

## 3. Results

**Figure 3:**
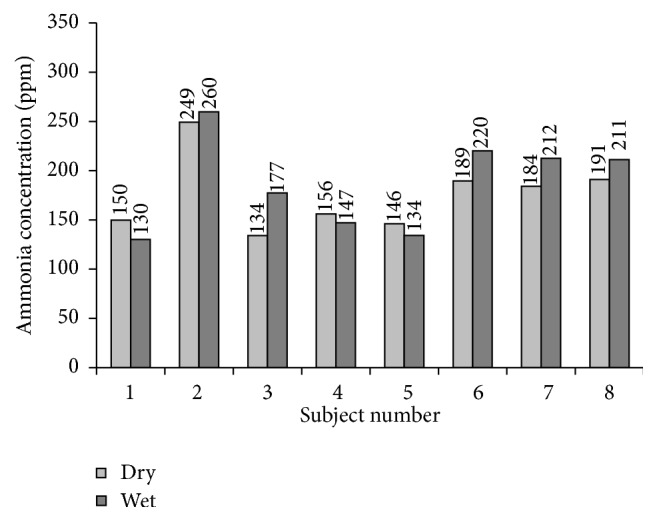
LTs for dry and wet ammonia vapor of all subjects.

Figure 3 shows LTs in parts per million for the wet and the dry conditions and for all subjects. The difference between the LT_wet_ (GM = 181 ppm; CI = 146–225 ppm) and the LT_dry_ (GM = 172 ppm; CI = 145–203 ppm) did not reach significance (*P* = 0.199). Both thresholds strongly correlated with each other (*r*_P_ = 0.88;* P* = 0.004). Across both conditions the average LT was 176 ppm (CI = 157–199 ppm).

**Figure 4:**
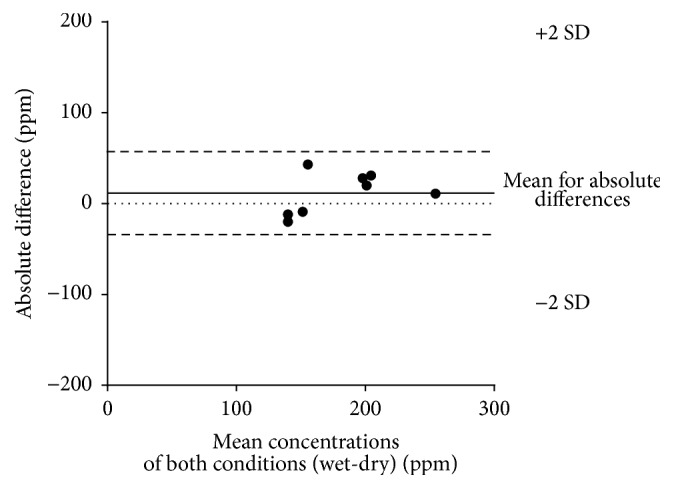
Comparison of LTs for wet and dry ammonia vapor. Differences between LT_wet_ and LT_dry_ plotted against the mean concentration according to Bland and Altman [18]. The continuous line shows the mean difference and the dashed line shows the ±2 SD for the differences.

The Bland-Altman plot shows (Figure 4) that LT_wet_ and LT_dry_ demonstrated a mean difference of 12 ppm (SD = 23 ppm), suggesting slightly higher LTs for the wet condition. The limits of agreement (absolute difference, −33 and 56 ppm) are small and all differences between the thresholds lay within ±2 SD.

**Figure 5:**
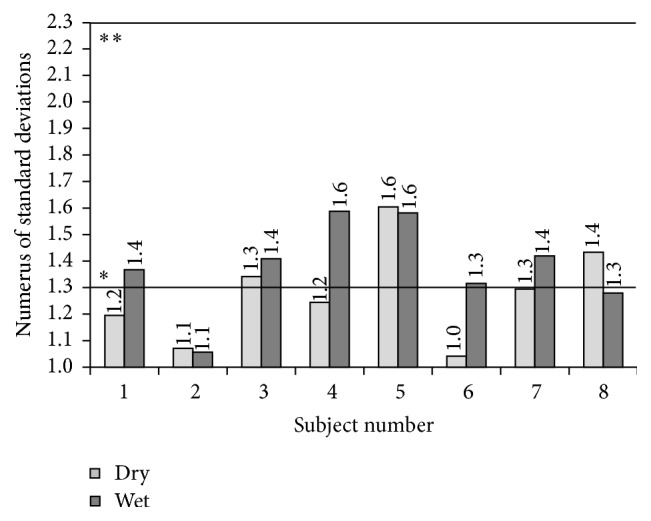
Numerus of all geometric standard deviations. The continuous line marked with one asterisk (*∗*) represents the average of all calculated numerus. The continuous line marked with two asterisks (*∗∗*) represents the required accuracy by CEN-criterion [8].

To ensure quality control, the threshold values from one day to another and within a single day should remain relatively unchanged. Therefore, to verify the repeatability of threshold measurements, which were conducted on three nonconsecutive days, the standard deviation of the logarithmic values (log 10) of all thresholds values (LT_wet_ and LT_dry_) was determined for each subject. According to [8] (page 34) the antilog of this standard deviation (called “numerus”) should be less than 2.3 to be sufficiently reliable. In our study, the average of all numerus was 1.3 (Figure 5).

## 4. Discussion

In a recent pilot study, we investigated the feasibility of a calibration gas generator to determine the odor and sensory irritation threshold of ammonia [16]. In that study, using a two-molar aqueous solution bottled in a transfusion bag (IAS GmbH, Germany) to generate different ammonia concentrations, we obtained LTs in the range of 109–208 ppm. However, the different water vapor content in each ammonia concentration may prove to be a disadvantage because they could have influenced the LTs. Therefore, in the current investigation we attempted to overcome this problem by using two different modifications to improve the method. The first modification addressed the time of presentation of the stimuli. We used triggered pinch valves to restrict the stimuli to two seconds. Furthermore, the pinch valves were intermittently closed in order to avoid emitting ammonia through the nosepieces. The time forced presentation method offered a further advantage by keeping the test conditions the same for all subjects. In contrast, the participants in our previous study were able to inhale the ammonia atmospheres through their noses for an arbitrarily long time. The second modification addressed the generation of ammonia atmospheres with only two different water vapor concentrations (dew point −12°C and 20°C).

Several recent studies calculated nasal LTs for ammonia and obtained values between 37 and 314 ppm. The different methodologies used in the various studies contributed to the observed variation in sensory irritation thresholds. Mean LTs were lower when assessed with static olfactometry (31.7 ppm; glass bottles), compared to those assessed with dynamic olfactometry (60.9 ppm; olfactometer) [15]. However, in another study using static olfactometry much higher LTs were assessed [6]. Compounds with high vapor pressure, such as ammonia, readily diffused into the gas phase under standard pressure and temperature conditions. As a result, the higher LTs might have been caused by the loss of vapor concentration between the consecutive runs, in particular from bottles containing lower concentrations [15]. Other important factors to consider that may influence LTs when using dynamic olfactometry include stimulus duration, interstimulus interval, and presentation procedures. It was previously shown that an increase in stimulus duration of about 2.5-fold compensates for a 2-fold decrease in concentration [13]. Furthermore, repetitive stimulation at short interstimulus intervals below 20 seconds may decrease the stinging effect of CO_2_ mediated by A_delta_-fibers (Hummel [19]). Nevertheless, the available studies assessing LTs for ammonia vapor show that similar methods result in significantly different LTs and different methods in similar LTs. Dynamic olfactometry, together with stimulus durations of about 10 seconds, and the velopharyngeal closure breathing technique resulted in nasal LTs between 37 and 67 ppm [13] and 167 and 179 ppm [14]. Both studies are slightly differed with respect to the presentation procedure, with the first study using a 2-up, 1-down staircase approach with varying stimulus durations and the second study using a two-alternative, forced-choice, up-down staircase approach with varying ammonia concentrations. In the present study, we used dynamic olfactometry, the ascending method of limits procedure, active sniffing, stimulus durations of 2 seconds, and interstimulus intervals of at least 30 seconds, which resulted in LTs between 172 ppm and 181 ppm. Even though our method is most comparable to that of Smeets et al. [15], the LTs obtained are rather similar to those of Petrova et al. [14] and those obtained in a previous study (109–208 ppm) where the stimulus duration was uncontrolled [16].

The calculations of the numerus of the geometric standard deviation of each sensory irritation threshold according to CEN [8] represent the exactness of the method used. In our study, the averaged numerus laid at 1.3 and is much less than 2.3 as requested by CEN-criterion. The criteria were established in economic terms, and thus reliable odor thresholds can be carried out in field trials typically with eight test persons. In our experiments, the inter- and intraindividual variability of the sensory irritation thresholds were very small and successfully fulfilled the required criteria. Therefore, we are convinced that a test panel of eight subjects was sufficient to perform our assessment.

Further technical and methodological development and standardization of threshold assessment procedures might allow better comparison of sensory threshold values across laboratories. Then determination of the chemosensory effect range could be used in a broader scale, either to estimate the irritant (trigeminal) potency of substances for which only few data are available or to evaluate existing studies reporting on sensory irritation.

Using the example of ammonia, the range of the LTs is comparable to results from human experimental studies that revealed no irritative effects at 25 ppm [20] or rather 50 ppm [21–23]. They are also in accordance with an animal study where rats were continuously exposed to ammonia. Clinical examination revealed increased blinking rates at a concentration of 100 ppm and histopathological signs of irritation were visible at 250 ppm [24]. The data do not support a higher sensitivity of humans, but the existing studies point to adaptation effects but no clear dose-response relationship. This needs to be considered and creates some uncertainty.

The goal of this study was to compare LTs for ammonia under different humidity conditions. However, we did not observe significantly different lateralization thresholds for wet or dry ammonia vapor after testing eight healthy nonsmoking men and women between 35 and 50 years. Of course, further studies are required in order to examine if these results can be extrapolated to other subject groups (e.g., subjects with mild asthma or allergic rhinitis). Studies evaluating LTs with CO_2_, also a highly water soluble but acidic compound, report heightened sensitivity among females or younger individuals and individuals with allergic rhinitis or preexisting nasal inflammation [25]. However, mild to moderate asthmatic subjects did not exhibit greater sensitivity or reactivity towards ammonia vapor than healthy subjects [14].

## 5. Conclusion

In conclusion, the influence of different water vapor concentrations in the ammonia atmospheres on the sensory irritation threshold (lateralization thresholds, LTs) is insignificant. Therefore, LTs of ammonia in other setups should be nearly unaffected by the presence of different water vapor concentrations. The obtained LTs lie within the range previously reported; however, it is reasonable to assume that water vapor can influence the sensory irritation threshold of other substances. Consequently, studies focusing on other highly soluble compounds will show how well the current findings can be generalized.
